# Selection Shapes Transcriptional Logic and Regulatory Specialization in Genetic Networks

**DOI:** 10.1371/journal.pone.0150340

**Published:** 2016-02-29

**Authors:** Karl Fogelmark, Carsten Peterson, Carl Troein

**Affiliations:** Computational Biology and Biological Physics, Department of Astronomy and Theoretical Physics, Lund University, 223 62 Lund, Sweden; Clemson University, UNITED STATES

## Abstract

**Background:**

Living organisms need to regulate their gene expression in response to environmental signals and internal cues. This is a computational task where genes act as logic gates that connect to form transcriptional networks, which are shaped at all scales by evolution. Large-scale mutations such as gene duplications and deletions add and remove network components, whereas smaller mutations alter the connections between them. Selection determines what mutations are accepted, but its importance for shaping the resulting networks has been debated.

**Methodology:**

To investigate the effects of selection in the shaping of transcriptional networks, we derive transcriptional logic from a combinatorially powerful yet tractable model of the binding between DNA and transcription factors. By evolving the resulting networks based on their ability to function as either a simple decision system or a circadian clock, we obtain information on the regulation and logic rules encoded in functional transcriptional networks. Comparisons are made between networks evolved for different functions, as well as with structurally equivalent but non-functional (neutrally evolved) networks, and predictions are validated against the transcriptional network of *E. coli*.

**Principal Findings:**

We find that the logic rules governing gene expression depend on the function performed by the network. Unlike the decision systems, the circadian clocks show strong cooperative binding and negative regulation, which achieves tight temporal control of gene expression. Furthermore, we find that transcription factors act preferentially as either activators or repressors, both when binding multiple sites for a single target gene and globally in the transcriptional networks. This separation into positive and negative regulators requires gene duplications, which highlights the interplay between mutation and selection in shaping the transcriptional networks.

## Introduction

The living cell can be viewed as a decision-making system that needs to respond appropriately to a wide range of external and internal signals in order to survive and maximize its reproductive success. Interacting components such as genes and proteins form networks that controls the flow of information. Such biological networks can be described at different scales, ranging from communication between large functional modules down to biochemically detailed models that include e.g. protein modifications. Here we consider genetic networks where the nodes are genes connected by edges that represent transcriptional regulation [1].

At all scales, evolutionary pressures shape networks under constraints imposed by their function. Biological functions may require specific network architectures and logic; for instance, an oscillator cannot work without a negative feedback loop. Some solutions are favourable because of greater evolvability and/or mutational stability; for instance, the modularity of evolved networks may resemble that of their engineered counterparts [2].

The structure of evolved biological networks can be replicated *in silico* through a combination of selection and large-scale duplication events [3]. On the other hand, the importance of selection has been questioned on the grounds that frequent and largely neutral rewiring events are able to explain common features of evolved transcriptional networks [4, 5]. In this view, gene duplications and other large mutation events are drivers of the exploration of the vast space of possible networks, with selection acting as a guide.

To study the balance between selection and neutral evolution *in silico*, as well as the role of gene duplications and other mutations, we need a model of evolvable transcriptional networks, including both network topology and transcriptional dynamics. Gene expression levels can be modelled either as continuous or discrete variables, each with its own advantages. Describing transcriptional regulation in terms of logic rules that govern gene expression is straightforward when the networks are modelled as discrete systems using Boolean functions [6]. However, real gene expression is not an all-or-nothing process, and a continuous model gives a more accurate representation of the transcriptional dynamics. Even so, the regulation of a gene is easier to understand in a Boolean description. We will therefore discretize the continuous expression levels only when analyzing the transcriptional logic encoded in networks.

A continuous dynamical model for the binding of transcription factors (TFs) to gene regulatory regions was derived by Banzhaf in [7]. Starting from a simplistic description of DNA and binding motifs as sequences of bits, the expression of a gene was determined by the sequence mismatch between the TFs and two binding regions, one activating and one repressing. However, limiting the regulatory region to only two non-interacting binding sites severely restricted the possibilities for transcriptional logic in that model.

In order to generate transcriptional logic functions, Buchler *et al*. constructed a model with competitive and cooperative binding of TFs at nearby binding sites. The recruitment or blocking of RNA polymerase (RNAP) depended on the positions of bound TFs, leading to activation or repression of the gene [8]. Cooperative binding, which in nature occurs through several possible molecular mechanisms, favours network connectivity and rewiring [5]. Even though Boolean terminology was used to describe the rich set of logic generated by this binding model, the underlying rules were continuous functions of TF concentrations.

Inspired by this earlier work, we construct a dynamical model of transcriptional regulation with combinatorial interactions of TFs on the DNA, which allows us to grow and evolve networks to explore the effects of selection. The representation of genes as strings of bits is borrowed from Banzhaf [7], but we allow multiple binding sites so that we can derive complex logic from the interactions of TFs within the regulatory region, as suggested by Buchler *et al*. [8]. In addition, we formulate dynamics for the production and degradation of proteins.

To perform network selection, we need to compute a biologically relevant measure of fitness based on the dynamics of the system. Ideally, we would like to simulate an entire organism, but this is not feasible. Instead, as targets for the simulated evolution we choose two well-defined computational problems with the potential to generate a variety of large circuits. The first is a relatively simple artificial problem: the majority rule, a Boolean decision problem which can be expected to yield networks with few feedback loops and mostly positive regulation. The second is a more complex problem which is directly linked to biology: the circadian clock, whose inherent properties include oscillations, internal feedbacks and input from the environment [9]. Simple oscillators have previously been used as a target for evolution in more detailed models of transcriptional and posttranslational regulation [10, 11].

Even if neutral mutations dominate the evolutionary process, the effects of selection must in some way be reflected in the structure of biological networks. The aim of this work is to identify markers of biological function in transcriptional networks evolved under selection, as compared to neutrally evolved ones, at the level of individual genes and their connections. To address this issue, we investigate properties such as the degree of specialization in transcription factors towards either activation or repression. We identify properties of regulation in functional networks, validate the results against the transcriptional network of *E. coli*, and show that large-scale mutations are necessary for reproducing the observed separation between activators and repressors.

## Methods

**Fig 1:**
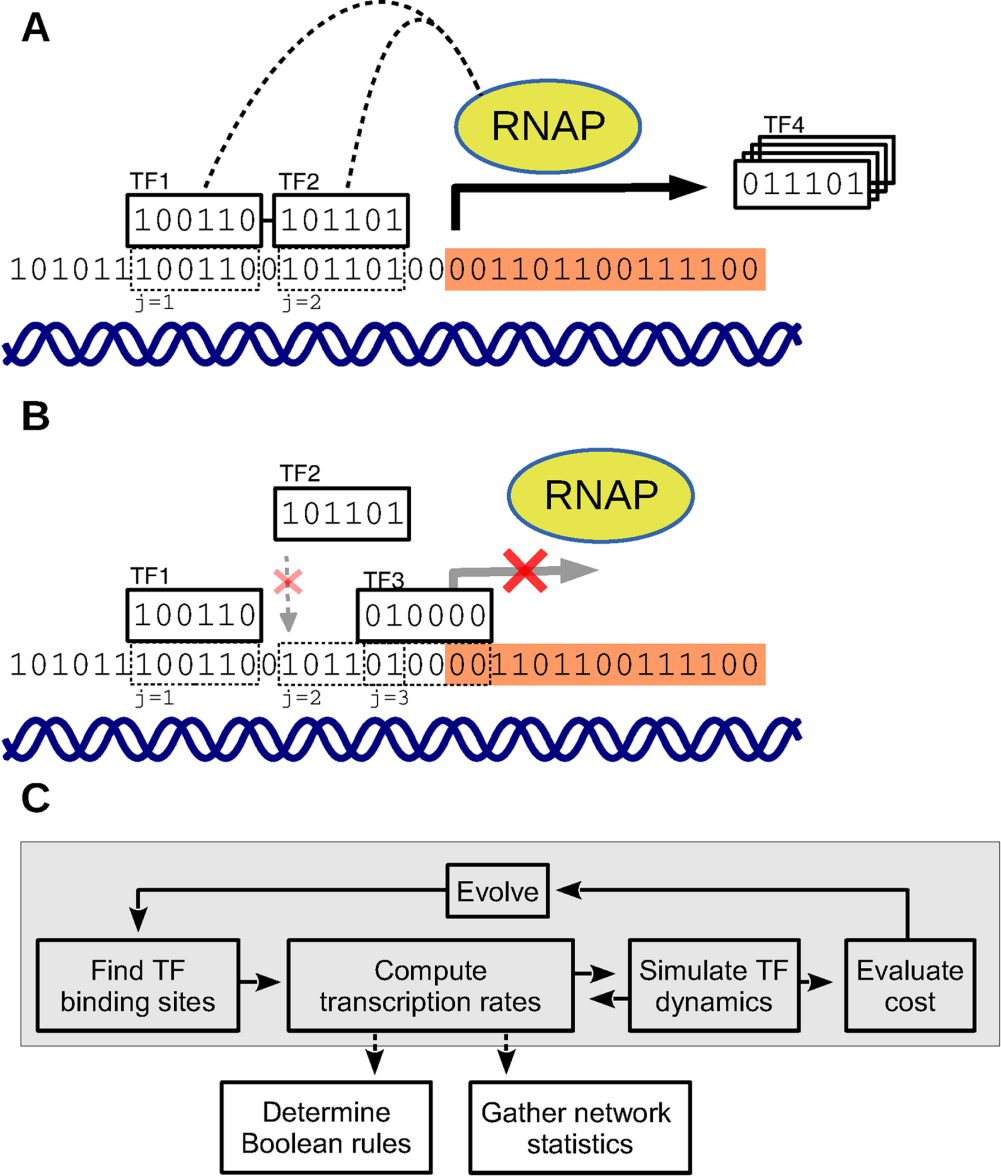
Transcriptional logic and networks derived from bitstrings. (A-B) A miniature example of possible interactions between 6-bit transcription factors at a 36-bit gene regulatory region. There are in this example binding sites for three different TFs (TF1, TF2, and TF3) which can bind to regulate the transcription rate of a fourth (TF4), whose properties are determined by information outside of the 36 bit regulatory region. The model computes the transcription rate as a mean over *all* possible states of TF–DNA binding, weighted by probability. In the notation of Eq (5), *A*_*g*_ = {1,2,3}. (A) Example of activation (*a* = {1,2} in Eq (5)): TF1 and TF2 are bound cooperatively (*C*_12_ = *e*^*β*^) and promote RNAP recruitment to the transcriptional start site. (B) Example of repression (*a* = {1,3}): When TF2 is not bound to site *j* = 2, TF3 is free to bind to site *j* = 3 and block the recruitment of RNAP, disabling transcription (*R*_3_ = 0). Binding by TF2 and TF3 is mutually exclusive (*C*_23_ = 0). Of all possible binding sites, half lead to repression when a TF blocks RNAP at or downstream of the transcriptional start site (orange region). In the full model, the TFs and DNA are longer than here (32 and 256 bits), and binding is possible also when sequences partially match (*H*_max_ = 6). (C) The simulation process. Each generation starts with the indexing of TF binding sites for all genes, which defines the transcriptional network. Transcription rates are computed according to Eq (5) and TF levels are updated according to Eq (6). The resulting dynamics are evaluated by a cost function whose value is used by the selection step of an evolutionary algorithm. When the evolution loop has completed after a number of generations, Boolean rules and other statistics are extracted for further analysis.

We have implemented a dynamical model of transcriptional regulation where proteins and regulatory regions are represented as sequences of bits, and transcription rates are determined by the interactions between TFs and DNA. An individual network consists of a variable number of genes, each represented as strings of bits. In the current model, all proteins are TFs, which may bind to the regulatory region of a gene to modify its expression, by either facilitating or inhibiting the recruitment of RNAP. A regulatory region may contain binding sites for many different TFs, giving rise to complex logic through the combinatorics of cooperative and mutually exclusive binding, as explained in the following section. This regulatory model is shown as miniature example in Fig 1A and 1B, together with an overview of the simulation process in Fig 1C.

### Transcriptional regulation

At the heart of the model, transcriptional regulation is derived from regulatory regions and binding motifs which are described as sequences of ones and zeroes. Typical binding motifs are about 5–20 basepairs in size, which motivates us to represent motifs by 32 bits to serve as 16 nucleotides, considering that DNA carries two bits of information per basepair. Regulatory regions are represented by 256 bits surrounding the transcriptional start site (TSS). Extending the regulatory regions to 512 bits produced similar results (S3 and S4 Figs), suggesting that the smaller size provides sufficient combinatorics at lower computational cost.

To form a network, every TF motif is compared with all positions along the DNA sequences, and binding is considered to occur where the Hamming distance (the number of mismatching bits) is below a threshold, *H*_max_ = 6 (with similar results at *H*_max_ = 8). A regulatory region may thus contain a large number of binding sites, and the same TF may bind to several sites. If a pair of binding sites have any overlap, they cannot be occupied simultaneously, and this exclusion limits the number of bound TFs at any given moment. For our choice of parameters, there are 256 − 32 + 1 = 225 possible binding sites and no more than 256/32 = 8 simultaneously bound TFs.

Transcriptional regulators may have properties that make them predominantly activating or repressing. Examples include the potent activator Gal4 in yeast [12] and the KRAB domain which is strongly associated with repression in eukaryotes [13]. To be able to investigate whether the separation of positive and negative regulation is a fundamental principle of gene networks, we based the model on the more flexible arrangement in *E. coli*, where the sign of TF regulation is primarily determined by the positions of binding sites relative to the TSS [14]. In the model, TFs that bind to the regulatory region upstream of the TSS act as activators to initiate or enhance transcription, whereas TFs that are located downstream of the TSS are assumed to block RNAP and act as repressors, disallowing any transcription of the gene. The TSS is located near the middle of the regulatory region, such that half of the possible binding sites are activating and half are repressing.

A TF may thus act as an activator for some genes and as a repressor for others, depending on where it finds a matching binding pattern on the DNA sequence. It may also regulate ambiguously, with binding sites of opposite signs in a single regulatory region.

Negative interactions are created by overlapping binding sites, but the model also includes cooperative binding between TFs at nearby binding sites, partly to capture the effects of complex formation and other protein–protein interactions. As motivated in the Buchler *et al*. model, two TFs that occupy closely spaced binding sites lower their binding energy by *β* = 3 *k*_*B*_*T* [8]. Presently, our model includes this cooperative interaction between all pairs of binding sites within a distance of 10 bits end-to-end, regardless of the identity of the TFs. This nonspecificity is a simplification aimed at capturing cooperative binding regardless of its mechanisms (cf. ref. [5]).

The transcription rate of gene *g* is determined by the recruitment of RNAP to its promotor region, which depends on the number of bound activating TFs, which in turn follows a distribution computed from the statistical weights of all possible binding states.

Given that *n* TFs are bound as activators, the probability of finding RNAP bound at gene *g* is

$$\begin{array}{c} P_{g}\left(n\right)=\frac{e^{-(b_{g}-\lambda n)}}{1+e^{-(b_{g}-\lambda n)}}, \end{array}$$ (1)

where the RNAP binding energy is lowered by *λ* = 3 *k*_*B*_*T* by each additional bound TF [8], and where the gene-specific ground state energy, −3 *k*_*B*_*T* < *b*_*g*_ < 9 *k*_*B*_*T*, allows for a wide range of basal transcription rates.

Assume that TF *i* can bind to site *j* with a mismatch of *H*_*ij*_ < *H*_max_ bits. The statistical weight of site *j* being occupied by any TF at time *t* is

$$\begin{array}{c} z_{j}\left(c\left(t\right)\right)=\sum_{i}\alpha e^{-\gamma H_{ij}}c_{i}\left(t\right), \end{array}$$ (2)

where the dimensionless TF concentration levels, ***c***(*t*), typically peak in the range 0.001 < *c*_*i*_ < 10 due to the dynamics (see Eq (6)). The binding affinity drops by $\gamma =\frac{1}{2}\ln10\;k_{B}T\approx 1.15\;k_{B}T$ for every mismatching bit, in agreement with experimentally measured mismatch energies of about 1–3 *k*_*B*_*T* per nucleotide [15]. The factor *α* = 1000 is required to convert the concentration levels into a realistic range of site occupancies.

The binding state *a* is defined as a set of occupied binding sites. Its statistical weight, *w*_*a*_, is a product of the weights of the individual sites and the interactions of all pairs of sites:

$$\begin{array}{c} w_{a}\left(c\left(t\right)\right)=\prod_{j\in a}z_{j}\left(c\left(t\right)\right)\prod_{j_{1}<j_{2}\in a}C_{j_{1}j_{2}}, \end{array}$$ (3)

where the contribution from the pair of sites *j*_1_, *j*_2_ is

$$\begin{array}{c} C_{j_{1}j_{2}}=\{\begin{array}{cc} 0, & \text{if}\;\text{exclusive}\\ e^{\beta }\approx 20, & \text{if}\;\text{cooperative}\\ 1, & \text{otherwise}. \end{array} \end{array}$$ (4)

Putting these pieces together, we arrive at an expression for the transcription rate of gene *g*, as a weighted mean over all binding states:

$$\begin{array}{c} T_{g}\left(c\left(t\right)\right)=\frac{\sum_{a\subset A_{g}}(P_{g}\left(|a|\right)w_{a}\left(c\left(t\right)\right)\prod_{j\in a}R_{j})}{\sum_{a\subset A_{g}}w_{a}\left(c\left(t\right)\right)}, \end{array}$$ (5)

where the sums run over all subsets of *A*_*g*_, the set of all identified binding sites for gene *g*, and |*a*| is the number of sites in subset *a*. There is no transcription if RNAP binding is blocked by any TF, as *R*_*j*_ is 0 if binding site *j* is in the repressing region and 1 otherwise.

As an implementation detail, the fact that *λ* in Eq (1) does not depend on the identity of the TF (see [8]) enabled us to implement Eq (5) with a dynamical programming algorithm that scales far better with the number of binding sites than a naive enumeration of all 2^|*A*_*g*_|^ states. This algorithm considers the sites in order and tracks the statistical weight of having up to *k* TFs bound, with the last being at site *j*; at worst it thus scales as 225 sites times 8 bound TFs, which is crucial when the transcription rate is evaluated at every time step. The algorithm can be extended to discrete TF-specific RNAP binding energies (*λ*), both positive and negative.

### Network dynamics

The scheme for transcriptional logic defines transcription rates for all genes, given the TF concentration levels, ***c***(*t*). To model the time development of these TF levels, transcription and translation are treated as a single step, a simplification motivated by the shorter typical timescale for turnover of mRNA compared to proteins [16]. The time derivative of the protein level for gene *g* is

$$\begin{array}{c} \frac{dc_{g}}{dt}=p_{g}T_{g}\left(c\left(t\right)\right)-d_{g}c_{g}\left(t\right), \end{array}$$ (6)

where the transcription rate is modified by a gene-dependent translation efficiency 1 < *p*_*g*_ < 10, which allows fine-tuning of protein levels. Protein degradation follows mass action kinetics at a protein-specific rate 0.1 < *d*_*g*_ < 10. We simulated the production and degradation of TFs deterministically, as a set of ordinary differential equations.

### Cost functions

The fitness of a network was evaluated in terms of its ability to produce a suitable response to a range of inputs. Two different systems were implemented using our model of transcriptional regulation.

**Fig 2:**
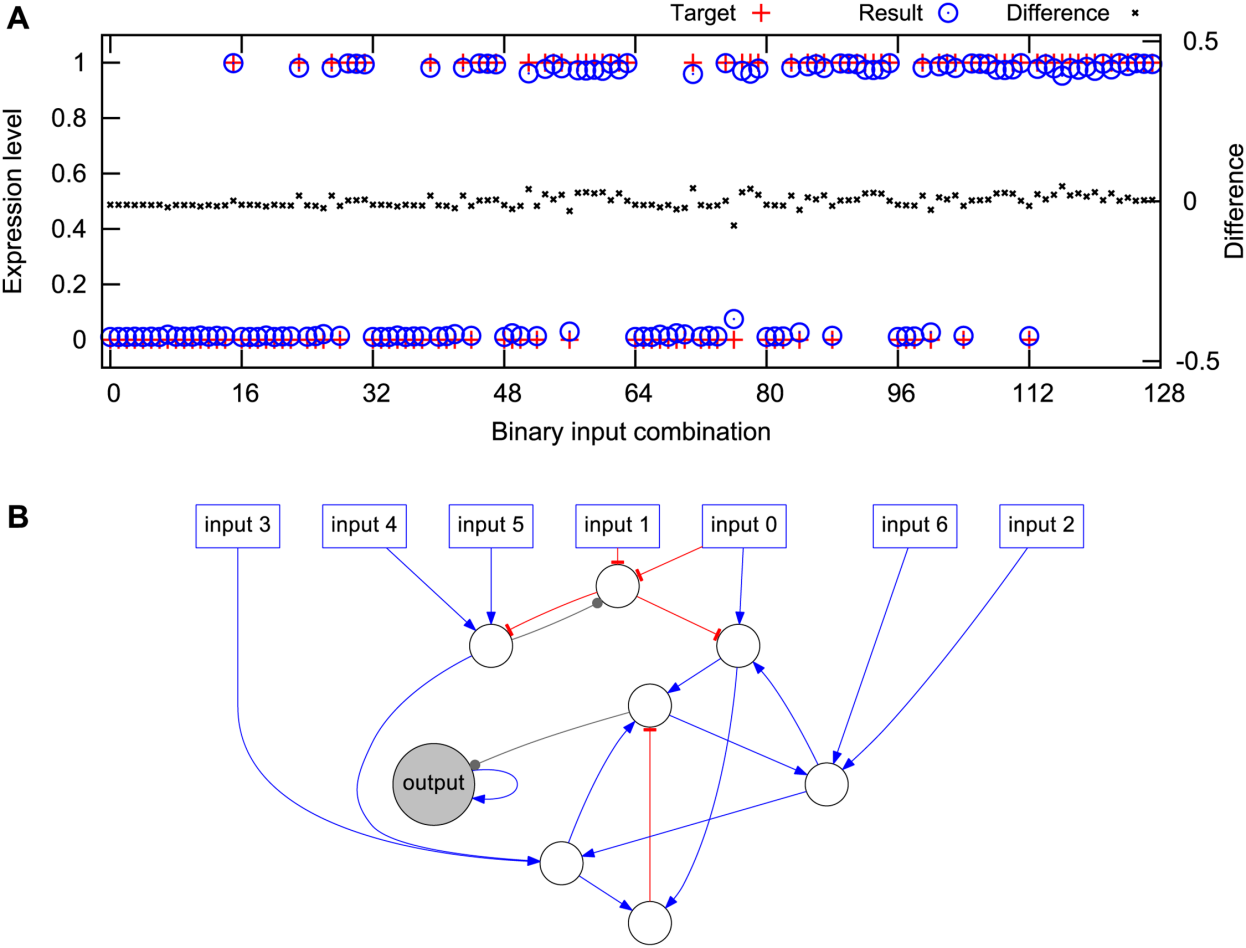
Evolving networks with majority function. Networks were selected for their ability to determine if the majority of seven binary inputs were on or off. (A) For all 128 possible input combinations, the network output (blue circles) should be as close as possible to the target (red dots), as measured by a cost function based on the deviations (black crosses, right hand *y*-axis). (B) The evolved network used to generate the output in (A). The input nodes (squares) take binary signals, and the output is the steady state level of the output node (grey). Blue edges with arrows represent activation and grey edges with circles represent ambiguous regulation. This example network was evolved for 2 ⋅ 10^6^ generations with a non-zero link cost in order to become suitably small for publication.

In the Boolean majority decision system seven TFs were used only as binary inputs, held at constant low or high levels (0 or 1, respectively). The task of the system was to determine whether a majority of the inputs were high or low; see Fig 2. A target profile was defined as 0 or 1 according to the majority of input bits, for each of the 2^7^ = 128 combinations of binary inputs. For each combination, we simulated the system for up to 480 time units, or until a fixed point was found. For each gene, the final expression levels were recorded and normalized to the same sum as the the target profile, to which they were then compared. The cost was defined as the mean square deviation of the best matching gene, normalized to 0 for a perfect match and 1 for a flat expression profile.

**Fig 3:**
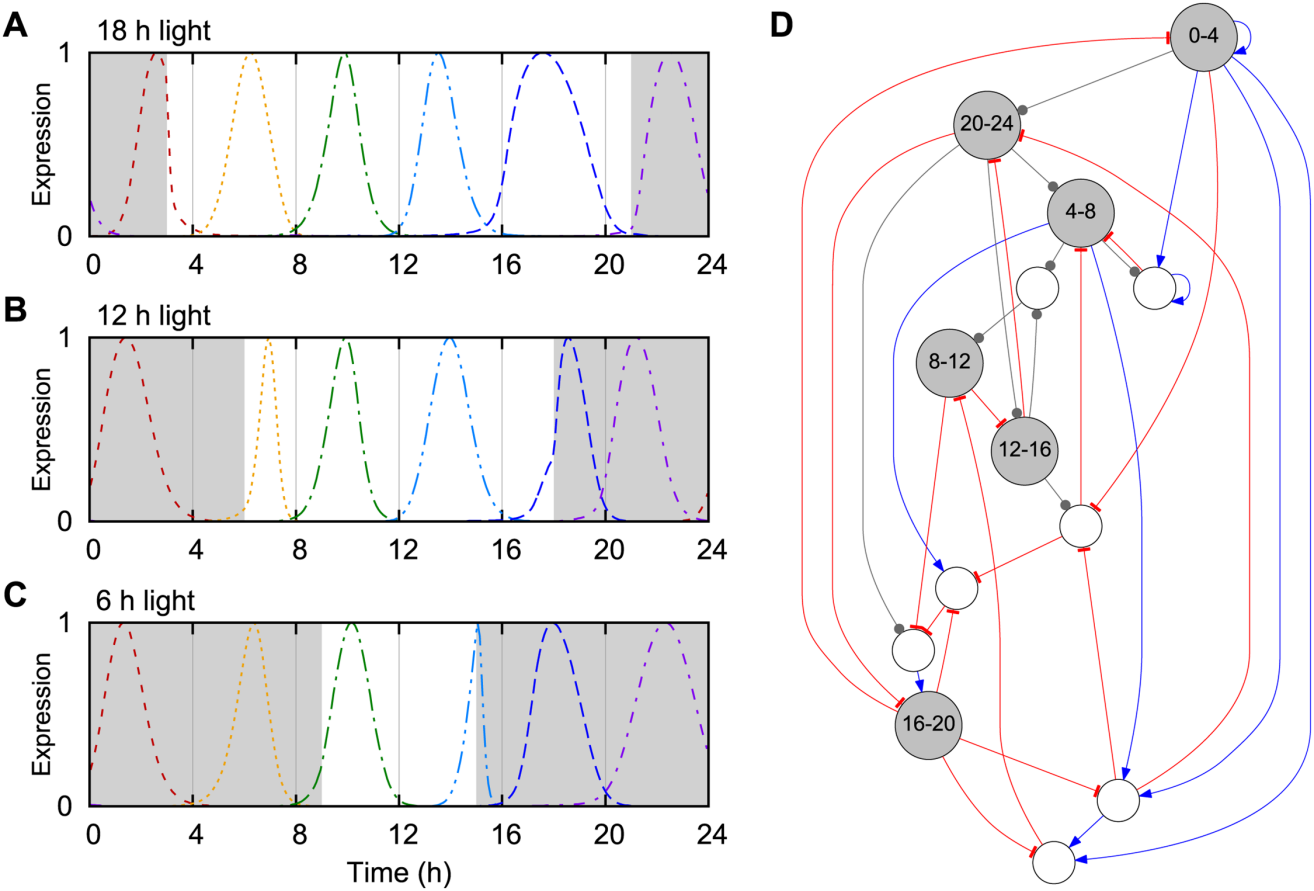
Evolving networks with clock function. Networks were selected for having each 4 h time window marked by the temporally focused expression of one TF. The six output TFs should be expressed at the correct time in 24 h periodic light conditions with 18 h (A), 12 h (B) and 6 h (C) of light. The coloured lines show the normalized expression levels of the output TFs, and the background is white/grey for light/dark. (D) The evolved network used to generate the output in panels A-C, with the output nodes (grey) labelled by time window. Light acts as input to all nodes through the protein degradation rates. Blue edges with arrows represent activation, red edges with bars represent repression and grey edges with circles represent ambiguous regulation. This example network was evolved for 2.5 ⋅ 10^6^ generations with a non-zero link cost in order to be suitably small for publication.

For the circadian clock system, we used a cost function that strives to focus the expression of a set of genes to specific times of the day. To encourage the emergence of a circadian clock, the network should find the correct timing of gene expression over a range of light conditions [17]. A large number of transcription and degradation rates are light-dependent in models of the plant clock [18]. We therefore simulated the input of light into the system through a 24 h periodic binary signal which selected between two independent sets of degradation rates for all TFs, *d*_*g*_ and $d_{g}^{\prime }$; see Fig 3.

For each light input, the network dynamics were run for a maximum of 20 days or until convergence to a limit cycle, with 6, 12 or 18 hours of light centred at noon. The expression level of each gene was integrated in six 4 h time windows over 24 h and normalized to a sum of 1. For each time window, the gene with the highest expression when averaged over the three different light conditions was chosen as the output in that window. The cost of the network was one minus the mean of the six output genes in their respective windows. Thus the cost function measured how well the system divided the 24 hours into six equal parts, reminiscent of the consecutively expressed PRR genes in the plant circadian clock [19].

### Evolution of fitness

To evolve the networks using an evolutionary algorithm, we defined mutations and crossover operations. Possible point mutations included alterations of the bitstrings for TFs and regulatory regions, the TF production rate and degradation rate (*p*_*g*_ and *d*_*g*_ in Eq (6)) and the affinity of RNAP for a specific promoter region (*b*_*g*_ in Eq (5)). Genes could also be deleted, and new genes were produced by duplication of a whole gene, from a recombination of a regulatory region and a TF, or, more rarely, *de novo* (which may be interpreted as an influx of genes into the system, e.g. from unrelated parts of the organism’s genome). The probabilities for the different types of mutations were chosen arbitrarily, and were not expected to greatly affect the results.

The initial network consisted only of a single randomized gene. Instead of imposing a small cost for each additional gene, which may impose an undue pressure on networks early in their evolution, we capped the total number of genes at 40, which is considerably more than the number of genes in, e.g., models of the *Arabidopsis* circadian clock. When studying the effects of evolution without gene duplication, we started the simulations with 40 random genes and disabled duplication events but allowed deletions and *de novo* gene creation.

For each generation of the evolutionary algorithm, we replaced the least fit individual out of a population of 20 networks. With 90% probability, the best of two randomly chosen individuals was duplicated and mutated. In the remaining cases, two parents were chosen in the same way (tournament selection) and used for crossover, where each gene of the offspring was picked from a random parent. After the final generation, the network with the lowest cost was saved. The networks were evolved for 7 ⋅ 10^4^ or 1 ⋅ 10^5^ generations for the majority rule or clock cost function, respectively. This process was iterated to create a sample of 100 independently evolved networks for each of our two cost functions. The resulting distributions in cost is shown in S1 Fig.

To make the networks more comparable with data on real transcriptional networks, we designed a pruning process to remove interactions that left the fitness nearly unchanged. The individual network links were sorted according to the fitness cost of removing them, and links were then removed one at a time until the total change in cost would have exceeded 0.01 (clocks) or 0.001 (majority rule).

### Neutrally evolved networks

To study the effects of selection, we generated networks without selection for function but with the same structural characteristics. The in-degree of a node (gene) was defined as the number of distinct TFs that bound to its regulatory region, and the out-degree was similarly defined as the number of distinct genes to which a TF bound. We constructed a cost function which compared two networks, such that a value of zero corresponded to the new network having the same number of nodes and edges, and the same distributions of in-degree and out-degree as the target network. Aside from this selection towards similar structure, the networks were allowed to evolve neutrally, using the same mutation mechanisms as the functional networks. From each functional network, we created 5 neutrally evolved networks.

### Extracting Boolean rules

The interactions of TFs that bind to a gene regulatory region result in a multivariate function, with input and output levels that need to be discretized if we are to extract a Boolean representation of the transcriptional logic rule. To build the truth table of a *k*-input rule, we applied all 2^*k*^ combinations of high and low input TF levels, where “low” was defined as 0 and “high” as the peak concentration level of the respective input TF in the dynamics. The gene was considered to be on for combinations where it reached at least half of its peak transcription rate observed in the dynamics, and off otherwise.

Many of the possible Boolean truth tables describe rules that do not depend on all of their inputs. This is a general problem when discretizing expression levels: TFs that only weakly affect the transcription rate may not be sufficient to push the output across the binarization threshold in any of the input conditions. For the analysis of Boolean rules, we removed all such “unused” inputs.

## Results

As described in Methods, we evolved functional networks with selection either for performing the Boolean majority rule (Fig 2) or for circadian clock function (Fig 3), with and without gene duplications as a possible mutation step, and compared these networks with structurally similar but non-fuctional networks created by neutral evolution. The functional networks showed considerable variation in fitness, with gene duplications improving the rate of convergence towards the selection target for the same number of generations (S1 Fig). As expected, selection for network function led to an enrichment in strong TF–DNA binding relative to random sequences, particularly for circadian clocks (S2 Fig).

### Low ambiguity of transcriptional regulation

A TF may act as an activator at one binding site and as a repressor at another, even within the same regulatory region. We refer to this case as ambiguous regulation of the target gene. The *E. coli* transcriptional network database RegulonDB includes information on this level, with nearly all TF–DNA interactions described as either activating or repressing. Disregarding a small number of binding sites with unknown or dual function, we could thus compare the prevalence of ambiguous regulation between the model and the *E. coli* data.

**Fig 4:**
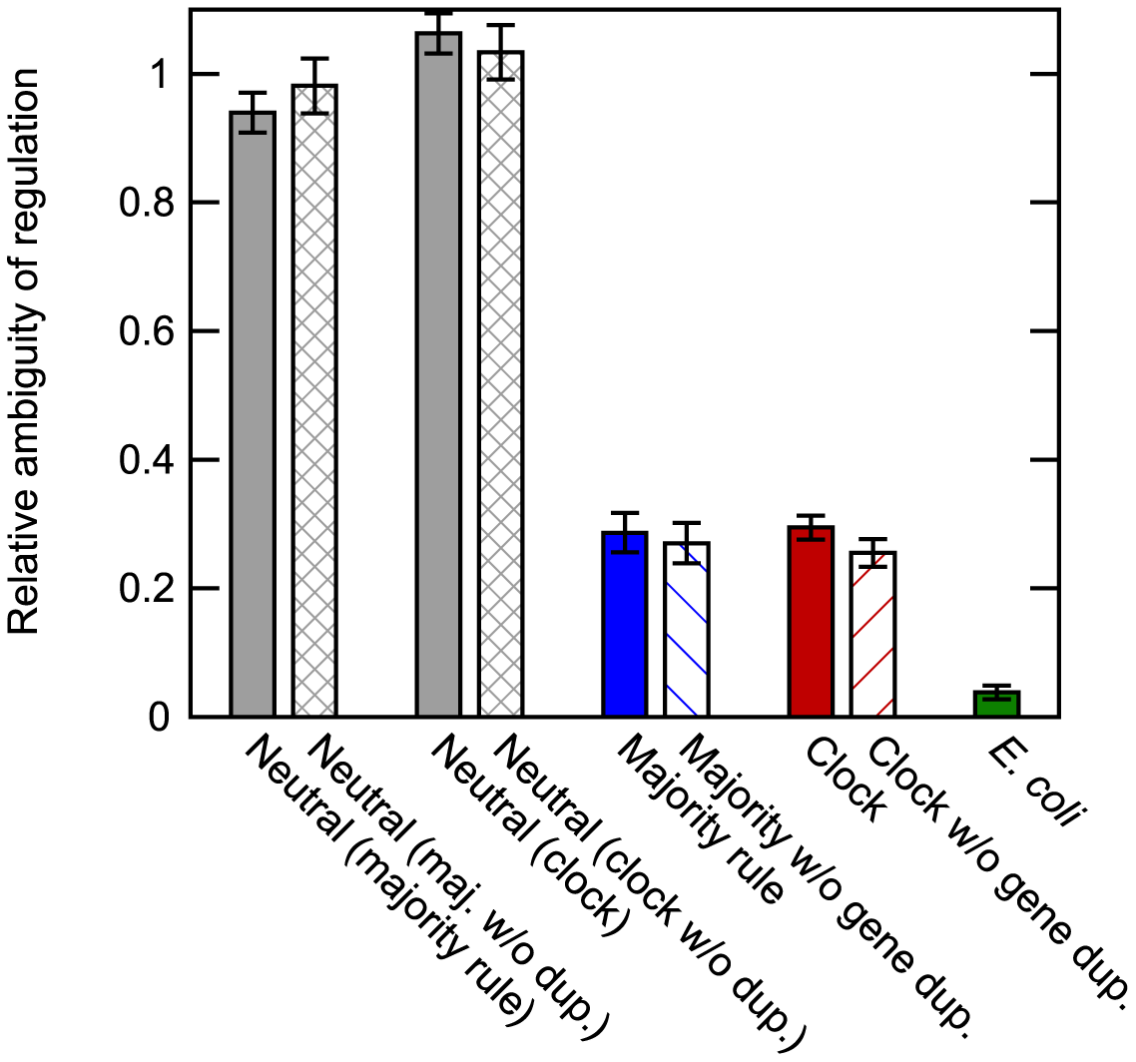
Prevalence of ambiguous gene regulation. The probability of a TF having exactly one activating and one repressing binding site in a regulatory region, relative to the null hypothesis that the sign of regulation is independent between sites (see main text). In neutrally evolved networks (gray), regulation was as ambiguous as expected by chance. In contrast, genes in functional evolved networks were predominantly regulated unambiguously, regardless of the network function (blue and red for majority rule and clock networks, respectively). Restricting the evolutionary paths by disabling gene duplications had little effect on this ambiguity (hashed bars). The model qualitatively predicted the situation in the transcriptional network of *E. coli* (green), where TFs almost always regulate their targets with a clearly defined sign. Binding site data were pooled from all networks; error bars indicate standard errors based on the total counts. See also S3 Fig, which explores the effects of some model parameter choices on the relative ambiguity.

Considering only cases where a TF bound to exactly two sites in a regulatory region, we defined *n*_++_, *n*_−−_ and *n*_+−_ as the number of activators, repressors and ambiguous regulators, respectively. If the sign of regulation is random, we expect that the ratio $n_{+-}/\left(2\sqrt{n_{++}n_{--}}\right)=1$. As shown in Fig 4, this relative ambiguity was indeed close to 1 in neutrally evolved networks. In networks evolved with selection, the ratio was much lower, between 0.2 and 0.3, regardless of the network function and whether gene duplications were allowed or not. This trend is in qualitative agreement with data from the *E. coli* network, where the relative ambiguity was only about 0.04.

The relative ambiguity was decreased by pruning unimportant links in the networks as described in Methods, whereas altering the TF–DNA binding cutoff to include many weaker binding sites (setting *H*_max_ = 8) had the opposite effect (S3 Fig). Strong and important links were thus associated with lower ambiguity, which could explain why we observed so few ambiguous interactions among those that have been found worthy of study and inclusion in RegulonDB.

### Binding site interactions

Depending on how they interact, every pair of binding sites in a regulatory region may be classified as competitive, cooperative or independent (Fig 1 and Eq (4)). A further division can be made into pairs of sites where either identical or different TFs bind. In the case of cooperative binding, these homogeneous and heterogeneous pairs of sites may represent binding by homo- and heterodimers, respectively.

**Fig 5:**
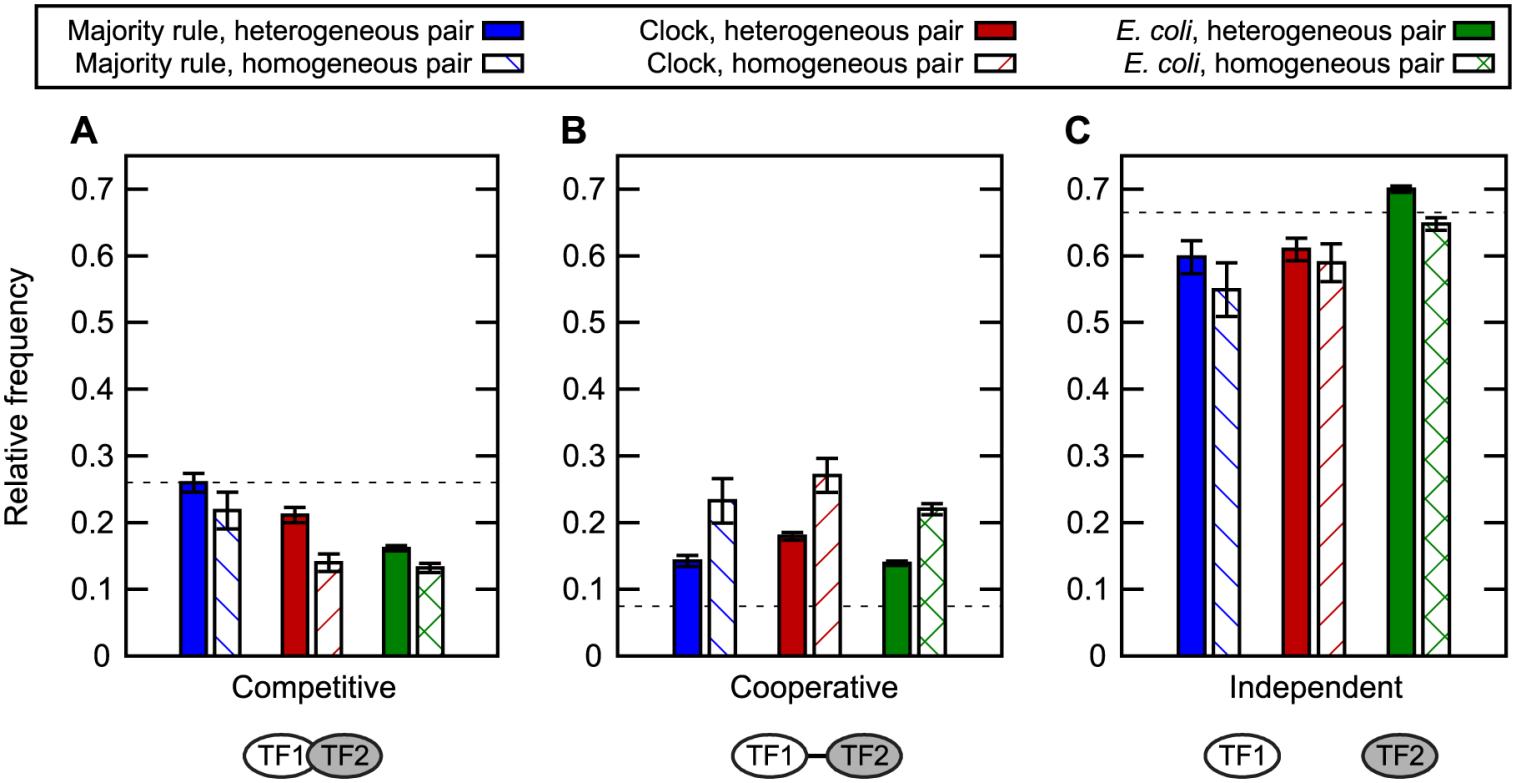
Pairwise interactions of TF binding sites. The fraction of pairs of TF binding sites that were (A) mutually exclusive because of competitive, overlapping binding, (B) cooperatively binding due to proximity, or (C) neither. Binding sites were defined as specific to one TF and may thus overlap completely on the DNA sequence. We further distinguished between homogeneous interaction (TF1 and TF2 are the same) and heterogeneous (TF1 and TF2 are different). Horizontal black dotted lines show the expectation values for random DNA and TF sequences. All pairs of binding sites (within the same regulatory region) were counted in 100 networks evolved as either majority rule (blue) or clock (red). *E. coli* data from RegulonDB [20] (green). Error bars indicate standard errors.

Regardless of the selection target used to evolve functional networks, competitive binding was considerably less likely between homogeneous pairs of binding sites than between heterogeneous ones (Fig 5A). It appears that networks have little use for components that directly counteract themselves. Conversely, cooperative binding was most likely between identical TFs (Fig 5B). Homodimer-like regulators were thus particularly favoured, but a comparison with the random expectation shows that cooperativity caused by heterodimer-like regulators was also significantly overrepresented.

To test these model predictions against data from a real transcriptional network, we collected statistics on the distance between midpoints of binding sites in *E. coli* from RegulonDB. For simplicity and comparability with the model, we classified sites within 16 basepairs as competitive and those within 32 basepairs as cooperative. As shown in Fig 5, a comparison between heterogeneous and homogeneous pairs of binding sites verified the model prediction that cooperative binding is preferentially associated with homodimers.

### Dominant sign of regulation

When a TF could bind to multiple sites at a gene, the regulation of that gene was predominantly either activating or repressing. This preference for a clear sign of regulation at the level of individual genes does not necessarily mean that TFs regulate all their targets in equal direction. We hypothesized that TFs may be divided into activators and repressors by selection if such a division constitutes a design principle for successful transcriptional networks.

**Fig 6:**
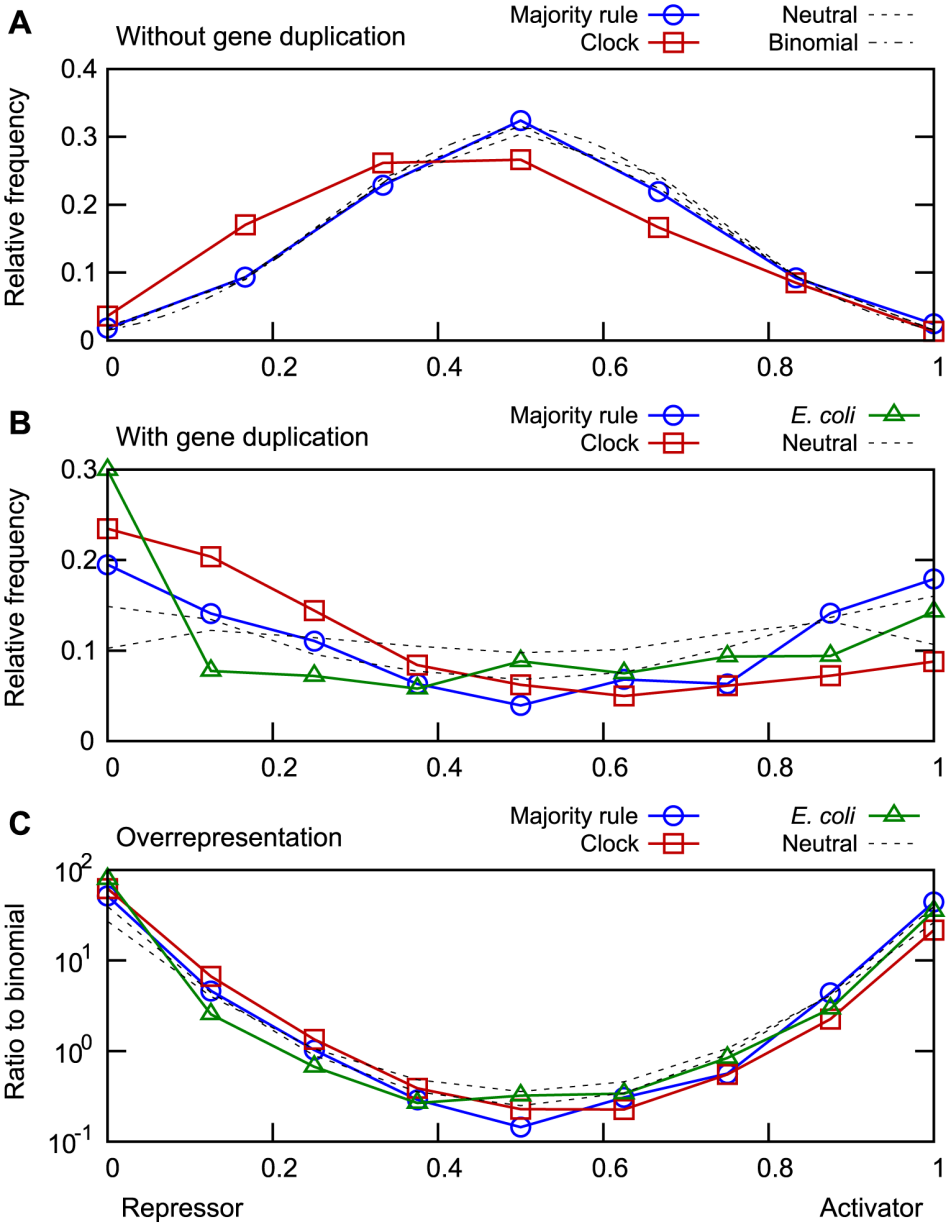
TF proclivity towards positive or negative regulation. For each TF, we computed the fraction of binding sites that enhanced rather than blocked transcription. The distribution of this quantity is presented for functional networks evolved either for majority rule or clock function (blue squares and red squares, respectively), compared with structurally similar neutrally evolved networks (black dashed lines) and with *E. coli* data [20] (green triangles) where applicable. (A) Without gene duplication, both functional and neutrally evolved networks followed the binomial distribution expected when individual target genes are randomly regulated (black dot-dashed line). A small bias towards negative regulation was observed in clock networks. (B) With gene duplications, TFs separated into mostly activating or repressing. The same pattern was observed in *E. coli*. (C) The relative overrepresentation of positive/negative regulators in functional networks, shown as the ratio between the data from panel B and the expectation for random networks. The number of pure activators or repressors was twentyfold to hundredfold higher than expected by chance, both in the simulated networks and in *E. coli*. The graphs are based on all TFs with at least 6 (A) or 8 (B-C) binding sites, with rebinning applied to those with additional sites. The lower limit in (A) is due to a scarcity of highly connected TFs in the majority rule networks; aside from statistical noise, the number of bins does not affect the shape of the curves. Data from 100 functional and 500 neutrally evolved networks of each kind.

We define the activator-repressor status of a TF as the fraction of a TF’s individual binding sites that are classified as activating. When all binding sites for a TF are considered, the expectation with random DNA and TF sequences is that the activator-repressor status follows a binomial distribution; it is rare to see mostly activation or repression by chance (Fig 6A).

The activator-repressor status of TFs in networks evolved neutrally without gene duplication was found to closely follow the expected binomial distribution. Similar results for networks selected for function indicated that selection did not encourage the separation of TFs into activators and repressors. The only deviation from the random expectation was a small bias towards negative regulation in clock networks (Fig 6A).

When gene duplication events were allowed, selection for either of the two network functions produced networks where the TFs were clearly separated into activators and repressors (Fig 6B). Neutral evolution produced similar results, which suggested that the separation between activators and repressors was not selected for, but rather a consequence of evolution following paths opened up by gene duplications. The results from the simulations were remarkably similar to data from *E. coli*. A majority of the TFs still had some binding sites of opposing signs, but pure activators and pure repressors were far more abundant than expected by chance (Fig 6C).

As before, clock networks showed a preference for negative regulators, while in the *E. coli* data we could see an overrepresentation of TFs without any activating interactions; these may represent inherent inhibitors of transcription.

### Transcriptional logic

The function of a transcriptional network is determined not only by its structure but also by the logic rules that govern the expression of its genes. These rules are defined by the binding and mutual interactions of TFs, and the transcriptional logic is thus constrained: only a subset of the Boolean functions can practically be realized [8]. As we have shown, our model predicts that the pairwise interactions between TFs follow similar patterns in networks evolved to perform different tasks. However, we would expect the resulting logic to differ.

The transcription rates, which are described by Eq (5) as continuous functions of the TF concentrations levels, were discretized into Boolean functions based on typical expression levels observed in the dynamics of the system. This procedure, which is explained in further detail in Methods, works only for functional networks; the neutrally evolved networks lack meaningful dynamics, and the resulting rules would depend strongly on arbitrary assumptions about expression levels. Hence, we have only compared Boolean rules extracted from functional evolved networks.

**Fig 7:**
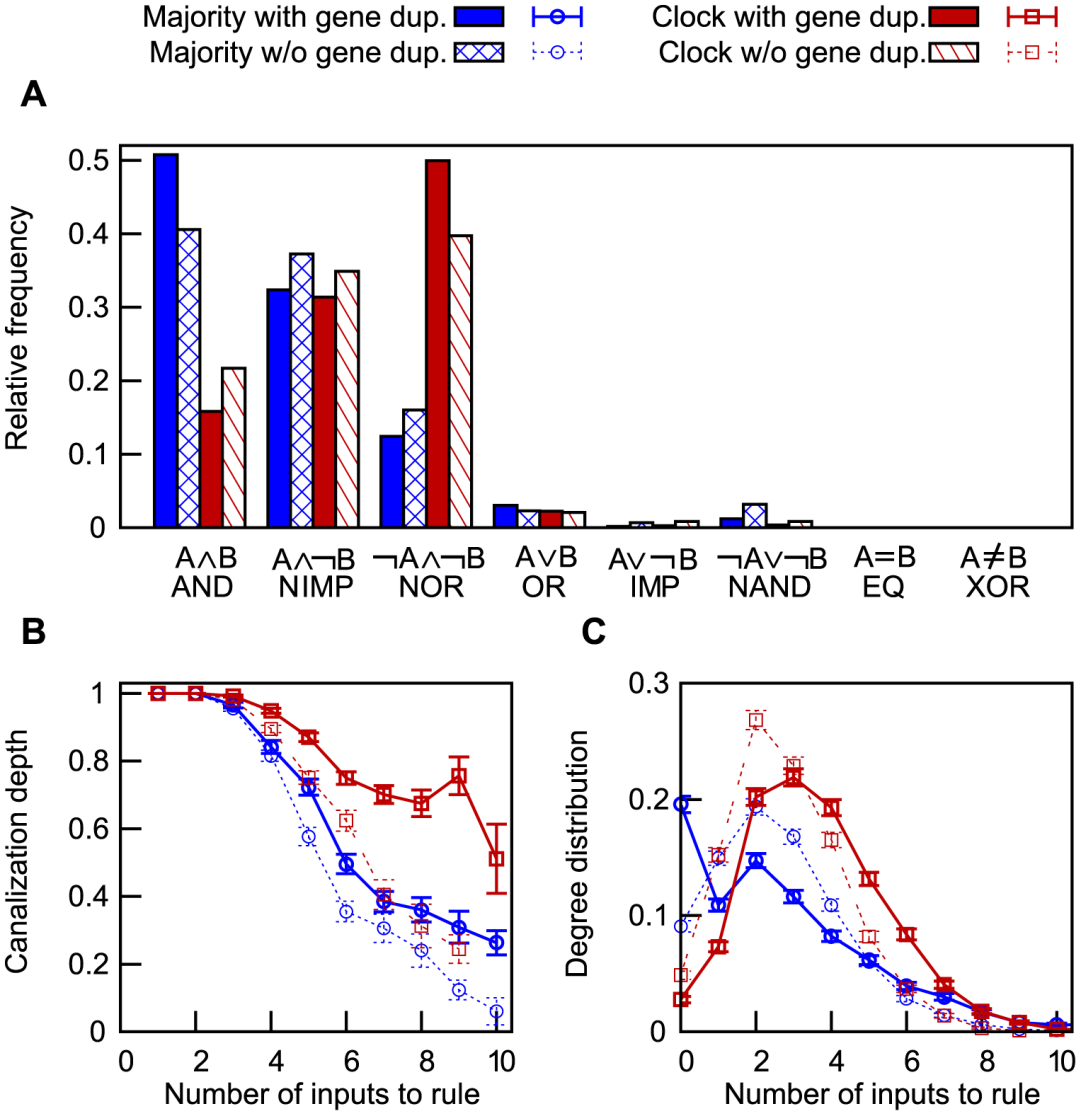
Transcriptional logic of evolved networks. Comparison between the logic rules in majority rule networks (blue) and clock networks (red), evolved with (solid) or without (hashed/dashed) gene duplications. (A) The relative frequencies among the eight rules that have only two inputs. (B) The structure of logic rules with up to 9 inputs, computed as the mean *nested canalizing depth* [21] normalized by the number of inputs. (C) The degree distribution of panel B. All three panels indicate that network function is a major factor in determining the distribution of transcriptional logic rules. The transcription rates, which were modelled as continuous functions of TF concentration levels, were discretized into Boolean logic rules as decribed in Methods. Data from 100 networks of each kind.

Binary Boolean rules such as AND and OR accounted for about 20% of the roughly 3000 multivariate rules extracted from each ensemble of networks (Fig 7C). The distribution of these rules is shown in Fig 7A, from which it can be seen that the presence or absence of gene duplication had little effect on the types of rules formed by rewiring of the transcriptional regulation. In contrast, the selection target strongly affected the number of rules of the most common types.

The most common logic rules were AND-like functions that tie together the presence of activators and the absence of repressors. The corresponding OR-like functions were relatively uncommon, accounting only for a few percent of the total. None of the evolved networks contained any cases of EQ or XOR, though tests with random sequences showed that such rules are possible to express in this model (not shown). The six binary rules that were realized are all *canalizing*, i.e., they have at least one input value that renders the other inputs irrelevant for determining the output. When the canalizing input is set to its non-canalizing value, the rule may be canalizing on additional inputs. Rules that are defined completely by such recursion are referred to as *nested canalizing*. These occur frequently in biology and generally lead to stable network dynamics [22].

A majority of the genes were regulated by more than two TFs. To characterize the resulting Boolean functions, we determined their *nested canalizing depth*. This number is maximal for nested canalizing rules such as *a* ∧ (*b* ∨ (*c* ∧ ¬*d*)) but zero for non-canalizing rules [21]. Two patterns emerged, one concerning biological function and one concerning evolutionary mechanisms: Clocks make use of more canalization than networks that solve the majority rule problem, and gene duplications favour the emergence of canalizing rules; see Fig 7B.

## Discussion

By applying *in silico* evolution to a combinatorial model of transcriptional regulation, we have explored how mutation and selection together shape interactions between genes in transcriptional networks. We have compared two specific network functions with the transcriptional network of an entire organism, partly for practical reasons but motivated by the philosophy that grand principles should be generic and robust.

The model demonstrates that TFs separate into activators and repressors even when they carry no inherent predisposition towards either sign of regulation. There are two aspects to this. First, the regulation of an individual gene by a TF is mostly unambiguously positive or negative; ambiguous regulation is relatively rare in nature as well as in our simulations of functional networks. Presumably, ambiguity leads to weak interactions that are unlikely to persist. Second, the model reproduces the observed broad separation of TF function into predominantly activating or repressing, but only in simulations of evolution that include gene duplications. These two points differ in one important regard: The former is specific to networks with functional dynamics, whereas the latter is also found in neutrally evolved networks. However, both are surprisingly insensitive to the choice of network function used as the target for selection.

In contrast, we found that the function of a network largely determined its distribution of logic rules, regardless of large-scale mutations such as gene duplications. Networks that were evolved to act as circadian clocks depended on negative interactions and distinctly canalizing logic rules. Furthermore, they were rich in cooperative binding and strong repression, in agreement with the view that oscillations are favoured by negative and nonlinear regulation of gene expression [23]. The importance of repression in circadian clocks is corroborated by modelling work in organisms such as *Arabidopsis thaliana* [24, 25]. In contrast, networks evolved to solve a simple decision problem made use of less canalizing logic and showed no bias towards repression.

Despite these differences, we observed strong similarities between all functional networks compared with neutrally evolved ones. This indicates that networks evolved with selection adhere to certain “design principles” for transcriptional regulation. TFs regulate their targets with less ambiguity and a higher incidence of cooperative binding than expected by chance, but the most striking feature is the polarization of the sign of regulation.

Four main conclusions can thus be drawn:

- The model produces testable hypotheses that agree with data from *E. coli*, such as the overrepresentation of cooperative binding among homogeneous pairs of binding sites (Fig 5).
- Selection leaves clear marks on gene regulation in functional networks; neutrally evolved networks do not regulate their genes in a coherent, unambiguous way (Fig 4).
- The evolutionary shortcuts created by gene duplications favour the approximate division of TFs into activators and repressors (Fig 6).
- The choice of target function for network selection has impact on the resulting logic rules, whereas the choice of allowed evolutionary paths appears to be of less importance; for the clock system, nested canalizing rules are dominant (Fig 7).

The importance of selection to the transcriptional regulation contrasts with earlier results on local structure by Kuo *et al*., who found that the prevalence of network motifs depended only on gene duplication and was unaffected by selection [3]. On the other hand, Kashtan *et al*. reported that selection did affect motif formation in modular information-processing networks compared with networks evolved only for modularity [26]. These conflicting results seem to be sensitive to model assumptions that affect the evolved non-functional networks. We expect greater robustness when conclusions are based on comparisons between functional networks, evolved either for different biological functions or using different mutational steps.

The effects of gene duplication on TF specialization requires further study. It appears that when target genes are duplicated, TFs retain their initial bias towards specialization despite subsequent rewiring of binding sites. Further work will be needed to clarify the robustness of this result. For instance, by tracking the evolutionary history of every binding site, we could quantify the extent and impact of the rewiring.

In the model presented here, transcriptional binding sites arise from a bit-based genotype, without prior assumptions about how the genes are connected. Network structure and function are created by the combinatorial interactions between transcriptional regulators. Models that include such a dynamical mapping from genotype to phenotype are well suited to mimic the complexity of natural systems and explore the mutation space to improve fitness [7]. The present model describes the core parts of transcriptional regulation in a simplified form, which is robust towards changes in model parameters (see e.g. S4 Fig). Depending on the context, the model could be extended to include explicit RNA and translation, as well as post-translational interactions such as protein complex formation. The model represents a simplified picture compared to the situation in *E. coli*, where repressors may bind upstream of the TSS [14], and to generic models that explicitly include longer-range interactions [8]. Future developments may thus include nonlocal interactions (DNA looping) as well as more TF-specific binding strengths and cooperative interactions.

## Supporting Information

S1 Fig Distribution of fitness in evolved networks. Final cost function values for networks evolved with or without gene duplication events, sorted by cost. (A) Majority rule networks evolved for 7 ⋅ 10^4^ generations. (B) Clock networks evolved for 1 ⋅ 10^5^ generations. Data from 100 simulations of each kind.
(EPS)

S2 Fig Distribution of TF–DNA binding strengths. The relative frequency of Hamming distances between TF binding motifs and DNA sequences, for all binding sites in networks evolved at binding strength cutoff *H*_max_ = 8. Data from 100 networks selected for function as the majority rule (blue circles) or circadian clock (red squares), either with or without gene duplications (thick solid and thin dashed, respectively). The enrichment in strong binding sites is due to selection; this is demonstrated by a comparison with 500 non-functional networks with similar structure (gray symbols and lines), which match the binomial distribution expected for random sequences (solid black line).
(EPS)

S3 Fig Prevalence of ambiguous gene regulation. The probability of a TF having exactly one activating and one repressing binding site in a regulatory region, relative to the probability under the null hypothesis that the sign of regulation is independent between sites. This figure expands on Fig 4 by including four model choices for neutrally evolved networks (gray), random regulatory regions and binding sequences (dark gray), majority rule networks (blue) evolved either normally, without gene duplications, without the pruning of unimportant interactions or with a larger regulatory region (512 bits), majority rule networks evolved with weaker binding mismatch cutoff (*H*_max_ = 8) with or without gene duplications or pruning, circadian clock networks for the same cases as the majority rule networks (red and orange) and data for *E. coli*. Unless otherwise stated, the model used *H*_max_ = 6 and 256-bit regulatory regions. As expected, *H*_max_ = 8 resulted in more ambiguous regulation due to a larger number of weak binding sites; this is reflected in the greater effect of pruning these networks. Increasing the size of the regulatory region leads to a decrease in ambiguity in the clock networks, which suggests that clocks sometimes use one negative and one positive binding site to implement their preferred cooperative negative regulation. Error bars indicate standard errors.
(EPS)

S4 Fig TF proclivity towards positive or negative regulation. This figure is identical to Fig 6A and 6B (*H*_max_ = 6, 256 bits), except that we here include data from networks with twice as large (512 bit) regulatory regions (dashed lines), or simulations with lower binding strength cutoff (*H*_max_ = 8, dot-dashed lines). The inclusion of weaker binding sites shifts the results towards the random expectation, but otherwise the results are qualitatively unchanged by these parameter changes. Data from 100 functional and 500 neutrally evolved networks of each kind.
(EPS)

S1 Data Data. This includes all data generated from the analysis of the networks.
(ZIP)
